# A Prospective Study to Identify the Prevalence of Impaired Glucose Tolerance in Patients With Liver Cirrhosis Using Oral Glucose Tolerance Test

**DOI:** 10.7759/cureus.38269

**Published:** 2023-04-28

**Authors:** Obul V Reddy, Vijaykumar G Warad

**Affiliations:** 1 General Medicine, Shri BM Patil Medical College and Hospital, BLDE University, Vijayapura, IND

**Keywords:** normal glucose tolerance, oral glucose tolerance test, liver cirrhosis, impaired glucose tolerance, diabetes mellitus

## Abstract

Introduction

Diabetes mellitus (DM) is a global health problem that may affect the prognosis of liver cirrhosis by interfering with the various metabolic functions of the body. Hence it is important to know the glycemic status of the patients with liver cirrhosis to anticipate and treat the complications associated with it, which in turn will help in the prognosis. The conventional methods may underestimate impaired glucose tolerance (IGT). Hence, this study was undertaken to identify IGT prevalent among liver cirrhosis patients using an oral glucose tolerance test (OGTT). The aim of our study was to identify the prevalence of IGT in non-diabetic liver cirrhosis patients using OGTT.

Materials and methods

This was a prospective cross-sectional study done in the Department of General Medicine at Shri BM Patil Medical College and Hospital, Vijayapura. After obtaining institutional ethical committee approval, a sample size of 85 liver cirrhosis patients between the age of 20-80 years who were not known cases of diabetes mellitus were selected from patients attending inpatient and outpatient departments at our hospital. The period of study was from January 2021 to June 2022. Patients were tested with OGTT, and the results were recorded.

Results

Our study found that IGT was more common in the age group of 40-49 years. All the patients included were males. We found that alcohol-induced liver cirrhosis patients had the maximum of IGT and DM. Our study observed a prevalence of 36.47% of patients with IGT. Our study in addition to IGT, found that DM was more common in 30-39 years, and 24.71% of patients were diagnosed with diabetes mellitus in the 85 patients included in our study.

Conclusion

We conclude that performing OGTT to find the prevalence of IGT and DM in liver cirrhosis patients will help in diagnosing DM and IGT, aiding in the improvement of the prognosis of the cirrhosis. The glycemic status of the patient may help in deferring the complications associated with poor glucose control. The incidence of potential complications of DM can be reduced by prompt identification and treatment.

## Introduction

Diabetes mellitus (DM) affects about 30% of cirrhotic patients. Whether type 2 diabetes, in the absence of obesity and hypertriglyceridemia, poses a risk for chronic liver disease is a topic of discussion today [1]. Since the late 1960s, it has been well-recognized that individuals with chronic liver disease frequently are found to have impaired glucose tolerance (IGT) and insulin resistance [2]. IGT and chronic liver disease are believed to interact and gradually hasten one another’s progression. Considering this, it may be challenging to distinguish the underlying causes of IGT in patients with chronic liver disease.

Although being the gold standard for diagnosing diabetes, the conventional fasting plasma glucose (FPG) criteria and hemoglobin A1C (HbA1C) readings may underestimate IGT. Because many of these patients have lower FPG readings or HbA1C, which conceal their IGT, it is common for the number of cirrhotic individuals with diabetes to be overestimated [3]. When a patient has a normal FPG but is suspected of having postprandial hyperglycemia, the oral glucose tolerance test (OGTT) is advised.

It is crucial to detect DM early because of the prognostic impact of DM in patients with liver cirrhosis. This study aims to find the prevalence of IGT in patients with liver cirrhosis by performing OGTT.

## Materials and methods

Study design

This was a prospective cross-sectional study conducted from January 2021 to August 2022 in the Department of General Medicine, BLDE Deemed to be University (DU), Shri BM Patil Medical College, Hospital and Research Center, Vijayapura, Karnataka, India, after obtaining the approval of Institutional Ethical Committee - BLDE (DU) (approval letter: IEC/No-09/2021). Non-diabetic patients between the age of 20 to 60 years who were diagnosed with liver cirrhosis using liver function tests and/or radiologically were included in the study. Patients with overt diabetes and pregnant patients were excluded from the study. Informed consent was obtained from all the patients included in the study.

Sample size

With an expected proportion of DM among patients with liver cirrhosis as diagnosed by the OGTT proportion rate of 35% [3], a sample size of 85 patients was needed for the study to have a 95% level of confidence and a 10% absolute precision. The formula used was $N= \frac{Z^{^{2}}-p\ast q}{d^{2}}$ where N is the number of patients, Z is the statistical level of significance, p is the rate of proportion, q=100-p, and d2 is the absolute error.

Procedure

Before obtaining the consent, all the patients included in the study were clearly informed of the study's purpose and procedures. The patients were instructed to fast for 12 hours the night before the OGTT. Before the OGTT, the baseline plasma glucose levels were determined using a glucometer, following which the patients received an oral glucose load of 75 grams (g) of anhydrous glucose in 300 milliliters (ml) of water, and after one- and two-hour time intervals, 3 ml of blood was collected into a blood collection tube with no additive. The collected blood sample was sent to the laboratory, where the sample was analyzed, and the plasma glucose levels were determined. According to the American Diabetes Association (ADA) guidelines, IGT was determined by a fasting blood sugar between 100 and 125 (milligram/deciliter) mg/dl and a two-hour glucose level between 140 and 199 mg/dl. Patients with a second-hour OGTT value above 200mg/dl were diagnosed with DM [4].

Statistical analysis

All collected data were entered into an Excel spreadsheet (Microsoft, Redmond, WA, USA), and statistical analysis was carried out using Statistical Package for Social Sciences (SPSS) version 20 (IBM Corp., Armonk, NY, USA). Tables were used to show the results, which were summarized as mean, median, standard deviation (SD), counts, and percentages (%). Unpaired t-tests and chi-square tests were used to determine the mean difference between the continuous and categorical variables. A P-value <0.05 was considered statistically significant.

## Results

**Figure 1:**
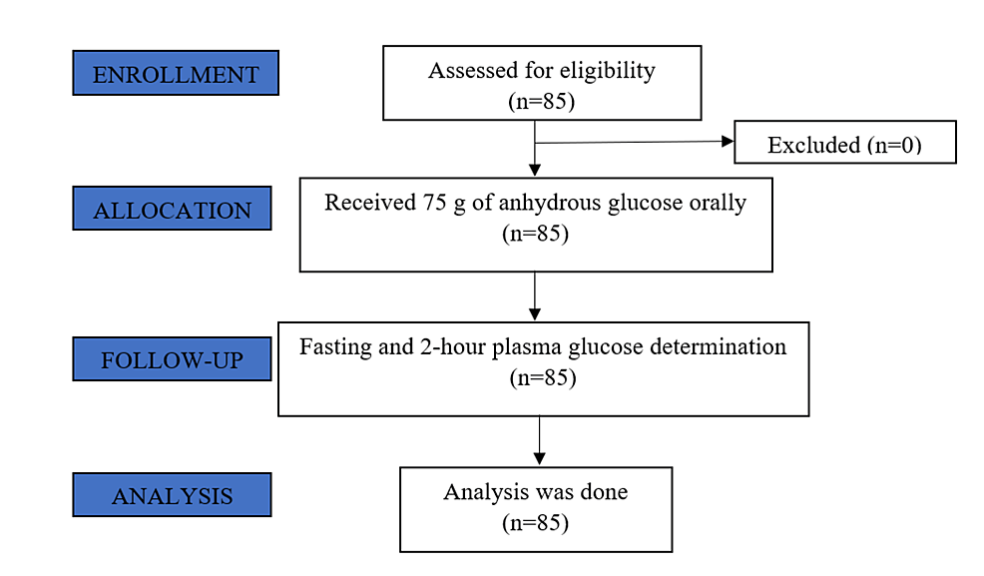
CONSORT Diagram CONSORT=Consolidated standards of reporting trials, n=number of patients

Figure 1 shows the Consolidated Standards of Reporting Trials (CONSORT) diagram depicting how patients were enrolled in our study.

Among those diagnosed with IGT, a maximum of 16 patients were found to be under the age of 40-49 years, eight patients were under the age of 30-39 years, and six patients were under the age of 50-60 years. Only one patient diagnosed with IGT belonged to the age group less than 30 years. Among those with DM, eight patients were under the age of 30-39 years, seven patients were under the age of 40-49 years, four patients were under the age of 50-60 years, and two patients were under the age of less than 30 years. The comparison of IGT, DM, and NGT between the different age groups was not statistically significant (P value=0.0640) (Table 1). 

**Table 1:** Age-wise distribution of the patients n=number of patients, IGT=Impaired Glucose Tolerance, DM=Diabetes Mellitus, NGT=Normal Glucose Tolerance

Age (Years)	IGT (n)	DM (n)	NGT (n)
< 30	1	2	10
30 – 39	8	8	8
40 – 49	16	7	9
50 – 60	6	4	6
Chi-square test	Chi-square=11.91, P value=0.0640		

All patients included in the study were males. Among the patients with alcohol-induced liver cirrhosis, the number of patients with IGT were 20, and those with DM were 12. In patients with hepatitis B-induced liver cirrhosis, 10 patients were found to have IGT, and nine patients were diagnosed to have DM. Only one patient had IGT in the patients with hepatitis C-induced liver cirrhosis. IGT was more common in all these etiological factors and was statistically significant (P value <0.05) (Table 2).

**Table 2:** Etiology of the liver cirrhosis n=number of patients, IGT=Impaired Glucose Tolerance, DM=Diabetes Mellitus, NGT=Normal Glucose Tolerance

Etiology	IGT (n)	DM (n)	NGT (n)	Total (n)	Chi-square test	P-value
Alcohol	20	12	32	64	12.683	0.00018
Hepatitis B	10	9	1	20	7.921	0.0191
Hepatitis C	1	0	0	1	2.008	0.3664

Out of the 85 patients included in the study, 36.47% were found to have IGT, and 24.71% were diagnosed with DM. The remaining 38.82% had normal glucose tolerance (NGT) (Table 3). This is statistically significant (P value<0.05).

**Table 3:** The prevalence of normal glucose tolerance, impaired glucose tolerance and diabetes mellitus IGT=Impaired glucose tolerance, DM=Diabetes Mellitus, NGT=Normal glucose tolerance

Variable	Number of patients	Percentage (%)	Odds ratio	P-value
IGT	31	36.47	1.273	0.048
DM	21	24.71		
NGT	33	38.82		

## Discussion

Despite the fact that DM and liver cirrhosis are concomitant conditions, the scope of the problem is typically understated. Due to the interaction of different factors in the etiology and pathophysiology of both diseases, the relationship between DM and liver cirrhosis is quite complex. Insulin resistance, steatosis, and DM have been linked to the hepatitis C virus (HCV) core protein, but overweight people with chronic HCV liver disease are more likely to develop these conditions than non-obese [3,5].

This study sought to prove that it is common practice to ignore and underappreciate the glycemic state in cirrhotic individuals. The outcome for these patients depends greatly on it. Numerous metabolic changes, primarily catabolic to muscle tissue, have been documented in liver cirrhosis. It is not yet understood how insulin resistance causes reduced glucose tolerance or overt diabetes mellitus. Receptor dysfunction probably exists in chronic liver disease that might be explained by the following factors such as modified membrane lipid composition and elevated free fatty acid levels, persistent insulin resistance, elevated plasma concentrations of hormones that block the action of insulin, including growth hormone, glucagon, catecholamines, and maybe cytokines, and deficiency in insulin-like growth factors I and II, liver-derived humoral factors with insulin-like action [6].

Impaired insulin sensitivity and subsequent changes in glucose metabolism (as shown by the high prevalence of insulin resistance and glucose intolerance) are unquestionably acquired in chronic liver diseases like cirrhosis. About 80% of cirrhotic individuals have glucose intolerance, 60% to 80% of them are insulin resistant, and 20% go on to develop overt diabetes mellitus. Long-standing theories suggest that chronic hyperinsulinemia causes or worsens insulin resistance [7].

Hepatogenous diabetes (HD) is the term used to describe diabetes mellitus related to liver cirrhosis. Despite the fact that it is widely acknowledged that liver cirrhosis is a diabetogenic condition, the American Diabetes Association (ADA) and the World Health Organization (WHO) do not recognize HD. Hemochromatosis, HCV, and alcohol-related cirrhosis are more frequently linked to HD than other etiologies [8].

Just a handful of research investigations have previously examined the prognostic value of diabetes in cirrhosis, and they discovered that diabetes was linked to a decreased survival rate [9,10]. In a French cohort analysis of 348 hospitalized patients with cirrhosis related to hepatitis C, DM was associated with shorter transplantation-free survival, regardless of the model for end-stage liver disease (MELD) score. Patients with a baseline MELD score of less than 10 had a significantly worse prognosis due to DM. Diabetes, on the other hand, had no effect on survival in patients with a baseline MELD score of more than or equal to 10 [11]. These results imply that the degree of liver disease conceals the harmful effects of diabetes and/or that in patients with high MELD scores.

It could be difficult to diagnose IGT and DM in cirrhotic people. In a study by Nishida et al., 23% of people with overt diabetes were shown to have normal fasting serum glucose levels, but their post-prandial blood sugar was more than 200 mg/dl [3]. Studies on small-sized series of patients showed that HbA1c measurements in cirrhotic individuals are not accurate [12-14]. In a study done by Lahousen et al. and Trenti et al., 40% of the HbA1c values in the cirrhotic non-diabetic patients were below the non-diabetic reference population. Moreover, patients with concomitant diabetes and cirrhosis also had HbA1c readings between 4 and 6% in the non-diabetic reference population. Only a few patients with cirrhosis and diabetes reported high HbA1c levels [14,15]. Thus, an OGTT is required to identify IGT and DM. 

Subclinical glucose intolerance estimated by OGTT is the second most significant prognostic factor next to serum albumin level. Garcia-Compean et al. did a study regarding impaired glucose tolerance that is subclinical as a predictor of death in liver cirrhosis and concluded that subclinical abnormal glucose tolerance has been associated with poor survival rate of patients with liver cirrhosis [1]. In our study, out of the 85 patients enrolled, 36.47% were found to have impaired glucose tolerance and 24.71% to have diabetes. Since the recent development of the treatment has remarkably improved the prognosis of cirrhosis, diabetic vascular complications in cirrhosis should not be overlooked. There is a strong correlation between liver cirrhosis and diabetes, and OGTT is a useful test for determining the glycemic state of these individuals.

Limitations

There are a few limitations in our study, such as the sample size, and also, the study included only males, so correlation with female patients was not established. The correlation between the etiology and development of IGT and DM was not studied.

## Conclusions

Our study found that patients with liver cirrhosis are more likely to have impaired glucose tolerance and diabetes mellitus. The OGTT is a trustworthy test, and it can be recommended as a routine examination of cirrhosis in addition to traditional liver function tests. OGTT should be employed as a routine test in patients with liver cirrhosis to diagnose IGT and DM, providing the chance to recognize and stop the consequences of hyperglycemia.
